# Robust Statistical Inference in Generalized Linear Models Based on Minimum Renyi’s Pseudodistance Estimators

**DOI:** 10.3390/e24010123

**Published:** 2022-01-13

**Authors:** María Jaenada, Leandro Pardo

**Affiliations:** Department of Statistics and Operation Research, Faculty of Mathematics, Complutense University of Madrid, Plaza Ciencias, 3, 28040 Madrid, Spain; mjaenada@ucm.es

**Keywords:** generalized linear model, independent and nonidentically distributed observations, minimum Rényi’s pseudodistance estimators, robust Wald-type test statistics for GLMs, influence function for GLMs, poisson regression model, 62F35, 62J12

## Abstract

Minimum Renyi’s pseudodistance estimators (MRPEs) enjoy good robustness properties without a significant loss of efficiency in general statistical models, and, in particular, for linear regression models (LRMs). In this line, Castilla et al. considered robust Wald-type test statistics in LRMs based on these MRPEs. In this paper, we extend the theory of MRPEs to Generalized Linear Models (GLMs) using independent and nonidentically distributed observations (INIDO). We derive asymptotic properties of the proposed estimators and analyze their influence function to asses their robustness properties. Additionally, we define robust Wald-type test statistics for testing linear hypothesis and theoretically study their asymptotic distribution, as well as their influence function. The performance of the proposed MRPEs and Wald-type test statistics are empirically examined for the Poisson Regression models through a simulation study, focusing on their robustness properties. We finally test the proposed methods in a real dataset related to the treatment of epilepsy, illustrating the superior performance of the robust MRPEs as well as Wald-type tests.

## 1. Introduction

Generalized linear models (GLMs) were first introduced by Nelder and Wedderburn [1] and later expanded upon by McCullagh and Nelder [2]. The GLMs represent a natural extension of the standard linear regression models, which enclose a large variety of response variable distributions, including distributions of count, binary, or positive values. Let $Y_{1},\ldots ,Y_{n}$ be independent response variables. The classical GLM assumes that the density function of each random variable $Y_{i}$ belongs to the exponential family, having the form

(1) $$f\left(y,\theta_{i},\phi \right)=\exp\left\{\frac{y\theta_{i}-b\left(\theta_{i}\right)}{a(\phi )}+c\left(y,\phi \right)\right\},$$

for i=1,…,n, where the functions a(ϕ),$b(\theta_{i})$ and $c\left(y,\phi \right)$ are known. Therefore, the observations are independent but not identically distributed, depending on a location parameter $\theta_{i},$i=1,…,n, and a nuisance parameter ϕ. Further, we denote by $\mu_{i}$ the expectation of the random variable $Y_{i}$ and we assume that there exists a monotone differentiable function, so called link function *g*, verifying

$$g\left(\mu_{i}\right)=x_{i}^{T}\beta ,$$

with $\beta =\left(\beta_{1},\ldots ,\beta_{k}\right)\in R^{k}$(k<n) the regression parameter vector. The k×1-vector of explanatory variables, $x_{i},$ is assumed to be nonrandom, i.e., the design matrix is fixed. Correspondingly, the location parameter depends on the explanatory variables $\theta =\theta \left(x^{T}\beta \right)$ the density function given in (1) can be written as $f_{i}\left(y,\beta ,\phi \right),$ empathizing its dependence of β and $x_{i}$.

The maximum likelihood estimator (MLE) and the quasilikelihood estimators were well studied for the GLMs, and it is well known that they are asymptotically efficient but lack robustness in the presence of outliers, which can result in a significant estimation bias. Jaenada and Pardo [3] revised the different robust estimators in the statistical literature and studied the lack of robustness of the MLE as well. Among others, Stefanski et al. [4] studied optimally bounded score functions for the GLM and generalized the results obtained by Krasker and Welsch [5] for classical LRMs. Künsch et al. [6] introduced the so-called conditionally unbiased bounded-influence estimate, and Morgenthaler [7], Cantoni and Ronchetti [8], Bianco and Yohai [9], Croux and Hesbroeck [10], Bianco et al. [11], and Valdora and Yohai [12] continued the development of robust estimators for the GLMs based on general M-estimators. Later, Ghosh and Basu [13] proposed robust estimators for the GLM, based on the density power divergence (DPD) introduced in Basu et al. [14].

There are not many papers considering robust tests for GLMs. In this sense, Basu et al. [15] considered robust Wald-type tests based on the minimum DPD estimator, but assuming random explanatory variables for the GLM. The main purpose of this paper is to introduce new robust Wald-type tests based on the MRPE under fixed (not random) explanatory variables.

Broniatowski et al. [16] presented robust estimators for the parameters of the linear regression model (LRM) with random explanatory variables and Castilla et al. [17] considered Wald-type test statistics, based on MRPE, for the LRM. Toma and Leoni–Aubin [18] defined new robustness and efficient measures based on the RP and Toma et al. [19] considered the MRPE for general parametric models, and constructed a model selection criterion for regression models. The term “Rényi pseudodistance” (RP) was adopted in Broniatowski et al. [16] because of its similarity with the Rényi divergence (Rényi [20]), although this family of divergences was considered previously in Jones et al. [21]. Fujisawa and S. Eguchi [22] used the RP under the name of γ-cross entropy, introduced robust estimators obtained by minimizing the empirical estimate of the γ-cross entropy (or the γ-divergence associated to the γ-cross entropy) and studied their properties. Further, Hirose and Masuda [23] considered the γ likelihood function to find robust estimation. Using the γ-divergence, Kawashima and Fujisawa [24,25] presented robust estimators for sparse regression and sparse GLMs with random covariates. The robustness of all the previous estimators is based on density power weight, $f{\left(y,\theta \right)}^{l},$ which gives a small weight to outliers observations. This idea was also developed by Basu et al. [15] for the minimum DPD estimator and was considered some years ago by Windham [26]. More concretely, Basu et al. [14] considered the density power function multiplied by the score function.

The outline of the paper is as follows: in Section 2, some results in relation to the MRPEs for GLMs, previously obtained in Jaenada and Pardo [3], are presented. Section 3 introduces and studies Wald-type tests based on the MRPE for testing linear null hypothesis for the GLMs. In Section 4, the influence function of the MRPE as well as the influence functions of the Wald-type tests are derived. Finally, we empirically examine the performance of the proposed robust estimators and Wald-type test statistics for the Poisson regression model through a simulation study in Section 5, and we illustrate its applicability with real data sets for binomial and Poisson regression.

## 2. Asymptotic Distribution of the MRPEs for the GLMs

In this Section, we revise some of the results presented in Jaenada and Pardo [3] in relation to the MRPE. Let $Y_{1},\ldots ,Y_{n},$ be INIDO random variables with density functions with respect to some common dominating measure, $g_{1},\ldots ,g_{n}$ respectively. The true densities $g_{i}$ are modeled by the density functions given in (1), belonging to the exponential family. Such densities are denoted by $f_{i}\left(y,\beta ,\phi \right)$ highlighting its dependence on the regression vector β, the nuisance parameter ϕ and the observation i, i=1,…,n. In the following, we assume that the explanatory variables $x_{i},$ are fixed, and therefore the response variables verify the INIDO set up studied in Castilla et al. [27].

For each of the response variables $Y_{i}$, the RP between the theoretical density function belonging to the exponential family, $f_{i}\left(y,\gamma \right),$ and the true density underlying the data, $g_{i},$ can be defined, for α>0 as

(2) $$R_{\alpha }\left(f_{i}\left(y,\gamma \right),g_{i}\right)=\frac{1}{\alpha +1}\log\left(\int f_{i}{\left(y,\gamma \right)}^{\alpha +1}dy\right)-\frac{1}{\alpha }\log\left(\int f_{i}{\left(y,\gamma \right)}^{\alpha }g_{i}\left(y\right)dy\right)+k,$$

where

$$k=\frac{1}{\alpha \left(\alpha +1\right)}\log\left(\int g_{i}{\left(y\right)}^{\alpha +1}dy\right)$$

does not depend on $\gamma ={\left(\beta^{T},\phi \right)}^{T}.$

We consider $\left(y_{1},\ldots ,y_{n}\right)$ a random sample of independent but nonhomogeneous observations of the response variables with fixed predictors $(x_{1},\ldots ,x_{n}).$ Since only one observation of each variable $Y_{i}$ is available, a natural estimate of its true density $g_{i}$ is the degenerate distribution at the the observation $y_{i}.$ Consequently, in the following we denote ${\hat{g}}_{i}$ the density function of the degenerate variable at the point $y_{i}.$ Then, substituting in (2) the theoretical and empirical densities, yields to the loss

(3) $$R_{\alpha }\left(f_{i}\left(y,\gamma \right),{\hat{g}}_{i}\right)=\frac{1}{\alpha +1}\log\left(\int f_{i}{\left(y,\gamma \right)}^{\alpha +1}dy\right)-\frac{1}{\alpha }\log f_{i}{\left(Y_{i},\gamma \right)}^{\alpha }+k.$$

If we consider the limit when α tends to zero we get

(4) $$R_{0}\left(f_{i}\left(y,\gamma \right),{\hat{g}}_{i}\right)=\lim_{\alpha ↓0}R_{\alpha }\left(f_{i}\left(y,\gamma \right),{\hat{g}}_{i}\right)=-\log f_{i}\left(Y_{i},\gamma \right)+k.$$

Last expression coincides with the Kullback–Leibler divergence, except for the constant k. More details about Kullback–Leiber divergence can be seen in Pardo [28].

For the seek of simplicity, let us denote

$$L_{\alpha }^{i}\left(\gamma \right)={\left(\int f_{i}{\left(y,\gamma \right)}^{\alpha +1}dy\right)}^{\frac{\alpha }{\alpha +1}},$$

and

$$V_{i}\left(Y_{i},\gamma \right)=\frac{f_{i}{\left(Y_{i},\gamma \right)}^{\alpha }}{L_{\alpha }^{i}\left(\gamma \right)}.$$

The expression (3) can be rewritten as

$$R_{\alpha }\left(f_{i}\left(y,\gamma \right),{\hat{g}}_{i}\right)=-\frac{1}{\alpha }\log\left(\frac{f_{i}{\left(Y_{i},\gamma \right)}^{\alpha }}{{\left(\int f_{i}{\left(y,\gamma \right)}^{\alpha +1}dy\right)}^{\frac{\alpha }{\alpha +1}}}\right)+k=-\frac{1}{\alpha }\log V_{i}\left(Y_{i},\gamma \right)+k.$$

Based on the previous idea, we shall define an objective function averaging the RP between all the the RPs. Since minimizing $R_{\alpha }\left(f_{i}\left(y,\gamma \right),{\hat{g}}_{i}\right)$ in γ is equivalent to maximizing $\log V_{i}\left(Y_{i},\gamma \right),$ we define a loss function averaging those quantities as

(5) $$T_{n}^{\alpha }\left(\gamma \right)=\frac{1}{n}\sum_{i=1}^{n}\frac{f_{i}{\left(Y_{i},\gamma \right)}^{\alpha }}{{\left(\int f_{i}{\left(y,\gamma \right)}^{\alpha +1}dy\right)}^{\frac{\alpha }{\alpha +1}}}=\frac{1}{n}\sum_{i=1}^{n}\frac{f_{i}{\left(Y_{i},\gamma \right)}^{\alpha }}{L_{\alpha }^{i}\left(\gamma \right)}=\frac{1}{n}\sum_{i=1}^{n}V_{i}\left(Y_{i},\gamma \right).$$

Based on (5), we can define the MRPE of the unknown parameter γ, ${\hat{\gamma }}_{\alpha },$ by

(6) $${\hat{\gamma }}_{\alpha }=\arg\max_{\gamma \in \Gamma }T_{n}^{\alpha }\left(\gamma \right),$$

with $T_{n}^{\alpha }\left(\gamma \right)$ defined in (5)

$$T_{n}^{0}\left(\gamma \right)=\frac{1}{n}\sum_{i=1}^{n}\log f_{i}\left(y_{i},\gamma \right)$$

at α=0. The MRPE coincides with the MLE at α=0, and therefore the proposed family can be considered a natural extension of the classical MLE.

Now, since the MRPE is defined as a maximum, it must annul the first derivatives of the loss function given in (5). The estimating equations of the parameters β and ϕ are given by

(7) $$\left\{\begin{array}{c} \frac{1}{n}\sum_{i=1}^{n}\frac{\partial V_{i}\left(Y_{i},\gamma \right)}{\partial \beta }=0_{k}\\ \frac{1}{n}\sum_{i=1}^{n}\frac{\partial V_{i}\left(Y_{i},\gamma \right)}{\partial \phi }=0. \end{array}\right.$$

For the first equation, we have

$$\begin{array}{cc} \frac{\partial V_{i}\left(Y_{i},\gamma \right)}{\partial \beta } & =\frac{1}{L_{\alpha }^{i}{\left(\gamma \right)}^{2}}\left\{\alpha f_{i}{\left(Y_{i},\gamma \right)}^{\alpha }\frac{\partial \log f_{i}\left(Y_{i},\gamma \right)}{\partial \beta }L_{\alpha }^{i}\left(\gamma \right)\right.\\ \; & \left.\;-\left[\alpha {\left(\int f_{i}{\left(y,\gamma \right)}^{\alpha +1}dy\right)}^{\frac{\alpha }{\alpha +1}-1}\int f_{i}{\left(y,\gamma \right)}^{\alpha +1}\frac{\partial \log f_{i}\left(y,\gamma \right)}{\partial \beta }dy\right]f_{i}{\left(Y_{i},\gamma \right)}^{\alpha }\right\}. \end{array}$$

The previous partial derivatives can be simplified as

$$\frac{\partial \log f_{i}\left(Y_{i},\gamma \right)}{\partial \beta }=\frac{Y_{i}-\mu_{i}}{Var\left(Y_{i}\right)g^{\prime }\left(\mu_{i}\right)}x_{i}=K_{1i}\left(Y_{i},\gamma \right)x_{i}$$

and

$$\frac{\partial \log f_{i}\left(Y_{i},\gamma \right)}{\partial \phi }=-\frac{\left(Y_{i}\theta_{i}-b\left(\theta_{i}\right)\right)}{a{\left(\phi \right)}^{2}}a^{\prime }\left(\phi \right)+\frac{\partial c\left(Y_{i},\phi \right)}{\partial \phi }=K_{2i}\left(Y_{i},\gamma \right).$$

See Ghosh and Basu [13] for more details. Now using the simplified expressions, we can write the estimating equation for β as

(8) $$\sum_{i=1}^{n}\frac{x_{i}}{L_{\alpha }^{i}\left(\gamma \right)}\left\{M_{i}\left(Y_{i},\gamma \right)-N_{i}\left(Y_{i},\gamma \right)\right\}=0_{k}$$

being

$$M_{i}\left(Y_{i},\gamma \right)=f_{i}{\left(Y_{i},\gamma \right)}^{\alpha }K_{1i}\left(Y_{i},\gamma \right)$$

and

$$N_{i}\left(Y_{i},\gamma \right)=\frac{f_{i}{\left(Y_{i},\gamma \right)}^{\alpha }}{\int f_{i}{\left(y,\gamma \right)}^{\alpha +1}dy}\int f_{i}{\left(y,\gamma \right)}^{\alpha +1}K_{1i}\left(y,\gamma \right)dy.$$

Subsequently, the estimating equation for ϕ, is given by

$$\begin{array}{cc} \frac{\partial V_{i}\left(Y_{i},\gamma \right)}{\partial \phi } & =\frac{1}{L_{\alpha }^{i}{\left(\gamma \right)}^{2}}\left\{\alpha f_{i}{\left(Y_{i},\gamma \right)}^{\alpha }\frac{\partial \log f_{i}\left(Y_{i},\gamma \right)}{\partial \phi }L_{\alpha }^{i}\left(\gamma \right)\right.\\ \; & \left.-\left[\alpha {\left(\int f_{i}{\left(y,\gamma \right)}^{\alpha +1}dy\right)}^{\frac{\alpha }{\alpha +1}-1}\int f_{i}{\left(y,\gamma \right)}^{\alpha +1}\frac{\partial \log f_{i}\left(y,\gamma \right)}{\partial \phi }dy\right]f_{i}{\left(Y_{i},\gamma \right)}^{\alpha }\right\}\\ \; & =\frac{1}{L_{\alpha }^{i}{\left(\gamma \right)}^{2}}\left\{\alpha f_{i}{\left(Y_{i},\gamma \right)}^{\alpha }\frac{\partial \log f_{i}\left(Y_{i},\gamma \right)}{\partial \phi }L_{\alpha }^{i}\left(\gamma \right)\right.\\ \; & \left.-\left[\alpha \frac{L_{\alpha }^{i}\left(\gamma \right)}{\int f_{i}{\left(y,\gamma \right)}^{\alpha +1}dy}\int f_{i}{\left(y,\gamma \right)}^{\alpha +1}\frac{\partial \log f_{i}\left(y,\gamma \right)}{\partial \phi }dy\right]f_{i}{\left(Y_{i},\gamma \right)}^{\alpha }\right\}. \end{array}$$

and thus, the estimating equation for ϕ is given by

(9) $$\sum_{i=1}^{n}\frac{1}{L_{\alpha }^{i}\left(\gamma \right)}\left\{M_{i}^{*}\left(Y_{i},\gamma \right)-N_{i}^{*}\left(Y_{i},\gamma \right)\right\}=0$$

being

$$M_{i}^{*}\left(Y_{i},\gamma \right)=f_{i}{\left(Y_{i},\gamma \right)}^{\alpha }K_{2i}\left(Y_{i},\gamma \right),$$

and

$$N_{i}^{*}\left(Y_{i},\gamma \right)=\frac{f_{i}{\left(Y_{i},\gamma \right)}^{\alpha }}{\int f_{i}{\left(y,\gamma \right)}^{\alpha +1}dy}\int f_{i}{\left(y,\gamma \right)}^{\alpha +1}K_{2i}\left(y,\gamma \right)dy.$$

Under some regularity conditions, Castilla et al. [27] established the consistency and asymptotic normality of the MRPEs under the INIDO setup. Before stating the consistence and asymptotic distribution of the MRPEs for the GLM, let us introduce some useful notation. We define

(10) $$\begin{array}{cc} S_{\alpha }^{i} & =\int f_{i}{\left(y,\beta ,\phi \right)}^{\alpha +1}dy\\ m_{jli}\left(\gamma \right) & =\frac{1}{\int f_{i}{\left(y,\gamma \right)}^{\alpha +1}dy}\int f_{i}{\left(y,\gamma \right)}^{\alpha +1}K_{ji}\left(y,\gamma \right)K_{li}\left(y,\gamma \right)dy,\\ m_{ji}\left(\gamma \right) & =\frac{1}{\int f_{i}{\left(y,\gamma \right)}^{\alpha +1}dy}\int f_{i}{\left(y,\beta ,\phi \right)}^{\alpha +1}K_{ji}\left(y,\gamma \right)dy,\\ l_{jli}\left(\gamma \right) & =\int \frac{f_{i}{\left(y,\gamma \right)}^{2\alpha +1}}{L_{\alpha }^{i}{\left(\gamma \right)}^{2}}\left(K_{ji}\left(y,\gamma \right)-m_{ji}\left(\gamma \right)\right)\left(K_{li}\left(y,\gamma \right)-m_{li}\left(\gamma \right)\right)dy, \end{array}$$

for all j,l=1,2 and i=1,…,n.

**Theorem** **1.** *Let $Y_{1},\ldots ,Y_{n}$ be a random sample from the GLM defined in (1). The MRPE ${\hat{\gamma }}_{\alpha }={\left({\hat{\beta }}_{\alpha }^{T},{\hat{\phi }}_{\alpha }\right)}^{T}$ is consistent and its asymptotic distribution is given by*

$$\sqrt{n}\Omega_{n}{\left(\gamma \right)}^{-\frac{1}{2}}\Psi_{n}\left(\gamma \right)\left(\left({\hat{\beta }}_{\alpha },{\hat{\phi }}_{\alpha }\right)-\left(\beta ,\phi \right)\right)\underset{n\to \infty }{\overset{L}{\to }}N\left(0_{k+1},I_{k+1}\right),$$

*where X denotes the design matrix, $I_{k}$ is the k-dimensional identity matrix and the matrices $\Psi_{n}$ and $\Omega_{n}$ are defined by*

$$\Omega_{n}\left(\gamma \right)=\frac{1}{n}\left(\begin{array}{cc} X^{T}D_{11}X & X^{T}D_{12}1\\ 1^{T}D_{12}X & 1^{T}D_{22}1 \end{array}\right),$$

$$\Psi_{n}\left(\gamma \right)=\frac{1}{n}\left(\begin{array}{cc} X^{T}\left(D_{11}^{*}-{\left(D_{1}^{*}\right)}^{T}D_{1}^{*}\right)X & X^{T}\left(D_{12}^{*}-{\left(D_{1}^{*}\right)}^{T}D_{2}^{*}\right)1\\ 1^{T}\left(D_{12}^{*}-{\left(D_{1}^{*}\right)}^{T}D_{2}^{*}\right)X & 1^{T}\left(D_{22}^{*}-{\left(D_{2}^{*}\right)}^{T}D_{2}^{*}\right)1 \end{array}\right),$$

*with*

$$D_{jk}=diag{\left(l_{jki}\left(\gamma \right)\right)}_{i=1,\ldots ,n,j,k=1,2}\;\;D_{jk}^{*}=diag{\left(m_{jki}\left(\gamma \right)\right)}_{i=1,\ldots ,n}$$

*and*

$$\;D_{j}^{*}=diag{\left(m_{ji}\left(\gamma \right)\right)}_{i=1,\ldots ,n},,j.k=1,2.$$

**Proof.**  The consistency is proved for general statistical models in Castilla et al. [27] and the asymptotic distribution of the MRPEs for GLM is derived in Jaenada and Pardo [3]. □

## 3. Wald Type Tests for the GLMs

In this section, we define Wald-type tests for linear null hypothesis of the form

(11) $$H_{0}:M^{T}\gamma =m\;\mathrm{vs}\;H_{1}:M^{T}\gamma \neq m$$

being $\gamma ={\left(\beta^{T},\phi \right)}^{T},$M a (k+1)×r full rank matrix and

(12) $$m={\left(m_{1},\ldots ,m_{r}\right)}^{T}$$

a *r*-dimensional vector (r≤k+1). If the nuisance parameter ϕ is known, as with logistic and Poisson regression, the matrix $M=L_{k\times r}.$ Additionally, choosing

$$M=\left(L_{k\times r},O_{1\times r}\right)$$

gives rise to a null hypothesis defined by a linear combination of the regression coefficients, β, with ϕ known or unknown. Further, the simple null hypothesis is a particular case when choosing M as the identity matrix of rank *k*,

$$H_{0}:\beta =\beta_{0}\;\mathrm{vs}\;H_{1}:\beta \neq \beta_{0}$$

with $m=\beta_{0}={\left(\beta_{1}^{0},\ldots ,\beta_{k}^{0}\right)}^{T}.$

In the following we assume that there exist a matrix $A_{\alpha }\left(\gamma \right)$ verifying

$$\lim_{n\to \infty }\Psi_{n}\left(\gamma \right)\Omega_{n}{\left(\gamma \right)}^{-1}\Psi_{n}\left(\gamma \right)=A_{\alpha }\left(\gamma \right).$$

**Definition** **1.** *Let ${\hat{\gamma }}_{\alpha }={\left({\hat{\beta }}_{\alpha }^{T},{\hat{\phi }}_{\alpha }\right)}^{T}$ be the MRPE of $\gamma ={\left(\beta^{T},\phi \right)}^{T}$ for the GLM. The Wald-type tests, based on the MRPE, for testing (11) are defined by*

(13) $$W_{n}\left({\hat{\gamma }}_{\alpha }\right)=n{\left(M^{T}{\hat{\gamma }}_{\alpha }-m\right)}^{T}{\left(M^{T}\Psi_{n}{\left({\hat{\gamma }}_{\alpha }\right)}^{-1}\Omega_{n}\left({\hat{\gamma }}_{\alpha }\right)\Psi_{n}{\left({\hat{\gamma }}_{\alpha }\right)}^{-1}M\right)}^{-1}\left(M^{T}{\hat{\gamma }}_{\alpha }-m\right).$$

The following theorem presents the asymptotic distribution of the Wald-type test statistics, $W_{n}\left({\hat{\gamma }}_{\alpha }\right).$

**Theorem** **2.** *The Wald-type test $W_{n}\left({\hat{\gamma }}_{\alpha }\right)$ follows asymptotically, under the null hypothesis presented in (11), a chi-square distribution with degrees of freedom equal to the dimension of the vector m in (12)*

*Under the null hypothesis given in (11) the asymptotic distribution of the Wald-type test statistics is a chi-square distribution with r degrees of freedom.*

**Proof.**  We know that

$$\sqrt{n}\left({\left({\hat{\beta }}_{\alpha }^{T},{\hat{\phi }}_{\alpha }\right)}^{T}-{\left(\beta^{T},\phi \right)}^{T}\right)\underset{n\to \infty }{\overset{L}{\to }}N\left(0_{k+1},A_{\alpha }{\left(\gamma \right)}^{-1}\right).$$

Therefore,

$$\sqrt{n}\left(M^{T}{\hat{\gamma }}_{\alpha }-m\right)=\sqrt{n}M\left({\hat{\gamma }}_{\alpha }-\gamma \right)\underset{n\to \infty }{\overset{L}{\to }}N\left(0_{k+1},M^{T}A_{\alpha }{\left(\gamma \right)}^{-1}M\right).$$

Now, the result follows taking into account that ${\hat{\gamma }}_{\alpha }$ is a consistent estimator of $\gamma_{0}.$ □

Based on the previous convergence, the null hypothesis in (11) is rejected, if

(14) $$W_{n}\left({\hat{\gamma }}_{\alpha }\right)>\chi_{r,\alpha }^{2}$$

being $\chi_{r,\alpha }^{2}$ the 100(1−α) percentile of a chi-square distribution with *r* degrees of freedom.

Finally, let $\gamma_{1}$ be a parameter point verifying $M^{T}\gamma_{1}\neq m,$ i.e., $\gamma_{1}$ is not on the null hypothesis. The next result establishes that the Wald-type tests given in (14) are consistent (see Fraser [29]).

**Theorem** **3.** *Let $\gamma_{1}$ be a parameter point verifying $M^{T}\gamma_{1}\neq m.$ Then the Wald-type tests given in (14) are consistent, i.e.,*

$$\lim_{n\to \infty }P_{\gamma_{1}}\left(W_{n}\left({\hat{\gamma }}_{\alpha }\right)>\chi_{r,\alpha }^{2}\right)=1.$$

**Proof.**  See Appendix A. □

**Remark** **1.** *In the proof of the previous Theorem was established the approximate power function of the Wald-type tests defined in (13),*

$$\pi_{W_{n}\left({\hat{\gamma }}_{\alpha }\right)}\left(\gamma_{1}\right)\approx 1-\phi_{N(0,1)}\left(\frac{1}{\sigma (\gamma_{1})}\left(\frac{\chi_{r,\alpha }^{2}}{\sqrt{n}}-\frac{W_{n}\left(\gamma_{1}\right)}{\sqrt{n}}\right)\right)$$

*where*

$$\sigma^{2}\left(\gamma_{1}\right)={\left(\frac{\partial l_{{\hat{\gamma }}_{\alpha }}\left(\zeta \right)}{\partial \zeta^{T}}\right)}_{\gamma =\gamma_{1}}A_{\alpha }{\left(\gamma_{1}\right)}^{-1}{\left(\frac{\partial l_{{\hat{\gamma }}_{\alpha }}\left(\zeta \right)}{\partial \zeta }\right)}_{\gamma =\gamma_{1}}$$

*and*

$$l_{{\hat{\gamma }}_{\alpha }}\left(\zeta \right)={\left(M^{T}{\hat{\gamma }}_{\alpha }-m\right)}^{T}{\left(M^{T}A_{\alpha }{\left(\zeta \right)}^{-1}M\right)}^{-1}\left(M^{T}{\hat{\gamma }}_{\alpha }-m\right).$$

From the above expression, the necessary sample size n for the Wald-type tests to have a predetermined power, $\pi_{0},$ is given by $n=[n^{*}]+1$, with

$$n^{*}=\frac{A+B+\sqrt{A(A+2B)}}{2l_{\gamma_{1}}^{2}\left(\gamma_{1}\right)}$$

*being*

$$A=\sigma^{2}\left(\gamma_{1}\right){\left(\phi^{-1}\left(1-\pi_{0}\right)\right)}^{2},\;B=2\chi_{r,\alpha }^{2}l_{\gamma_{1}}\left(\gamma_{1}\right)$$

*and [·] the integer part.*

In accordance with Maronna et al. [30], the breakdown point of the estimators ${\hat{\gamma }}_{\alpha }$ of a parameter γ is the largest amount of contamination that the data may contain such that ${\hat{\gamma }}_{\alpha }$ still gives enough information about γ. The derivation of a general breakdown points it is in general not easy, so it may deserve a separate paper where it may be jointly considered the replacement finite-sample breakdown point introduced by Donoho and Huber [31]. Although breakdown point is an important theoretical concept in robust statistics, perhaps is more useful the definition of breakdown point associated to a finite sample: replacement finite-sample break down point. More details can be seen in Section 3.2.5 of Maronna et al. [30].

## 4. Influence Function

We derive in this section the IF of the MRPEs of the parameters $\gamma ={\left(\beta^{T},\phi \right)}^{T}$ and Wald-type statistics based on these MRPEs, $W_{n}\left({\hat{\gamma }}_{\alpha }\right).$ The influence function (IF) of an estimator quantifies the impact of an infinitesimal perturbation in the true distribution of the data on the asymptotic value of the resulting parameter estimate (in terms of the corresponding statistical functional). An estimator is said to be robust if its IF is bounded. If we denote $G=\left(G_{1},\ldots ,G_{n}\right)$ the true distributions underlying the data, the functional $T_{\alpha }\left(G\right)$ and associated to the MRPE for the parameters γ is such that

$$\frac{1}{n}\sum_{i=1}^{n}R_{\alpha }\left(f_{i}\left(y,T_{\alpha }\left(G\right)\right),g_{i}\left(y\right)\right)=\min_{\gamma }\frac{1}{n}\sum_{i=1}^{n}R_{\alpha }\left(f_{i}\left(y,\gamma \right),g_{i}\left(y\right)\right).$$

The IF of a estimator is defined as the limiting standardized bias due to infinitesimal contamination. That is, given a contaminated distribution at the point $\left(y_{t},x_{t}\right)$, $G_{\varepsilon }=\left(1-\varepsilon \right)G+\varepsilon \Delta_{\left(y_{t},x_{t}\right)}$ with $\Delta_{\left(y_{t},x_{t}\right)}$ the degenerate distribution at $\left(y_{t},x_{t}\right)$, the IF of the estimator ${\hat{\gamma }}_{\alpha }$ in terms of its associated functional $T_{\alpha }\left(G\right)$ is computed as

$$\mathrm{IF}\left(\left(y_{t},x_{t}\right),T_{\alpha }\left(G\right)\right)=\lim_{\varepsilon \to 0}\frac{T_{\alpha }\left(G_{\varepsilon }\right)-T_{\alpha }\left(G\right)}{\varepsilon }.$$

In the following, let us denote $T_{\alpha }\left(G\right)=\left(T_{\alpha }^{\beta }\left(G\right),T_{\alpha }^{\phi }\left(G\right)\right),$ where $T_{\alpha }^{\beta }\left(G\right)$ and $T_{\alpha }^{\phi }\left(G\right)$ are the functionals associated the parameters β and ϕ, respectively. Then, they must satisfy the estimating equations of the MRPE given by

(15) $$\begin{array}{cc} \sum_{i=1}^{n}\frac{x_{i}}{L_{\alpha }^{i}\left((T_{\alpha }^{\beta }\left(G\right),T_{\alpha }^{\phi }\left(G\right))\right)}\left\{M_{i}\left(y_{i},\left(T_{\alpha }^{\beta }\left(G\right),T_{\alpha }^{\phi }\left(G\right)\right)\right)-N_{i}\left(y_{i},\left(T_{\alpha }^{\beta }\left(G\right),T_{\alpha }^{\phi }\left(G\right)\right)\right)\right\} & =0_{k}\\ \sum_{i=1}^{n}\frac{1}{L_{\alpha }^{i}\left((T_{\alpha }^{\beta }\left(G\right),T_{\alpha }^{\phi }\left(G\right))\right)}\left\{M_{i}^{*}\left(y_{i},\left(T_{\alpha }^{\beta }\left(G\right),T_{\alpha }^{\phi }\left(G\right)\right)\right)-N_{i}^{*}\left(y_{i},\left(T_{\alpha }^{\beta }\left(G\right),T_{\alpha }^{\phi }\left(G\right)\right)\right)\right\} & =0 \end{array}$$

where the quantities $L_{\alpha^{i}\left(\gamma \right)},M_{i}\left(y_{i},\gamma \right),N_{i}\left(y_{i},\gamma \right),M_{i}^{*}\left(y_{i},\gamma \right)$ and $N_{i}^{*}\left(y_{i},\mathrm{gamma}\right)$ are defined in Section 2. Now, evaluating the previous equation at the contaminated distribution $G_{\varepsilon }$, implicitly differentiating the estimating equations in ε and evaluating them at ε=0, we can obtain the expression of the IF for the GLM.

We first derive the expression IF of MRPEs at the $i_{0}-th$ direction. For this purpose, we consider the contaminated distributions

$$G_{i_{0},\varepsilon }=\left(G_{1},\ldots ,G_{i_{0}-1},\;G_{i_{0},\varepsilon },G_{i_{0}+1},\ldots ,G_{n}\right),$$

with $G_{i_{0},\varepsilon }=\left(1-\varepsilon \right)G_{i_{0}}+\varepsilon \Delta_{\left(y_{i_{0}},x_{i_{0}}\right)}.$ Here, only the $i_{0}$-th component of the vector of distributions is contaminated. If the true density function $g_{i}$ of each variable belongs to the exponential model, we have that

$$g_{i}\left(y\right)=\left\{\begin{array}{cc} f_{i}\left(y,\gamma \right) & i\neq i_{0}\\ \left(1-\varepsilon \right)f_{i}\left(y,\gamma \right)+\varepsilon \Delta_{\left(y_{i_{0}},x_{i_{0}}\right)}\left(y\right) & i=i_{0}. \end{array}\right.$$

Accordingly, we define

$$\gamma_{\varepsilon }^{i_{0}}=T_{\alpha }\left(G_{1},\ldots ,G_{i_{0}-1},G_{i_{0},\varepsilon },G_{i_{0}+1},\ldots ,G_{n}\right)$$

the MRPE when the true distribution underlying the data is $G_{i_{0},\varepsilon }.$ Based on Remark 5.2 in Castilla et al. [27] the IF of the MRPE at the $i_{0}-th$ direction with $\left(y_{i_{0}},x_{i_{0}}\right)$ the point of contamination is given by

$$\begin{array}{l} IF(\left(y_{i_{0}},x_{i_{0}}\right),T_{\alpha },G)={\left(\frac{\partial T_{\alpha }\left(G_{i_{0},\gamma_{\varepsilon }}\right)}{\partial \varepsilon }\right)}_{\varepsilon =0}\\ =\Psi_{n}{\left(\gamma \right)}^{-1}\frac{f_{i_{0}}{\left(y_{i_{0}},\gamma \right)}^{\alpha }}{\int f_{i_{0}}{\left(y,\gamma \right)}^{\alpha +1}dy}\left(\begin{array}{c} K_{1i}\left(y_{i_{0}},\gamma \right)-f_{i_{0}}{\left(y_{i_{0}},\gamma \right)}^{-\alpha }N_{i_{0}}\left(y_{i_{0}},\gamma \right)\\ K_{2i}\left(y_{i_{0}},\gamma \right)-f_{i_{0}}{\left(y_{i_{0}},\gamma \right)}^{-\alpha }N_{i_{0}}^{*}\left(y_{i_{0}},\gamma \right) \end{array}\right)\left(\begin{array}{cc} x_{i_{0}} & 0\\ 0 & 1 \end{array}\right). \end{array}$$

In a similar manner, the IF in all directions (i.e., all components of the vector of distributions are contaminated) has the following expression

$$\begin{array}{l} IF(\left(y_{1},x_{1}\right),\ldots ,\left(y_{n},x_{n}\right),T_{\alpha },G)={\left(\frac{\partial T_{\alpha }\left(G_{\gamma_{\varepsilon }}\right)}{\partial \varepsilon }\right)}_{\varepsilon =0}\\ =\Psi_{n}{\left(\gamma \right)}^{-1}\sum_{i=1}^{n}\left(\frac{f_{i}{\left(y_{i},\gamma \right)}^{\alpha }}{\int f_{i}{\left(y,\gamma \right)}^{\alpha +1}dy}\left(\begin{array}{c} K_{1i}\left(y_{i},\gamma \right)-f_{i}{\left(y_{i},\gamma \right)}^{-\alpha }N_{i}\left(y_{i},\gamma \right)\\ K_{2i}\left(y_{i},\gamma \right)-f_{i}{\left(y_{i},\gamma \right)}^{-\alpha }N_{i}^{*}\left(y_{i},\gamma \right) \end{array}\right)\left(\begin{array}{cc} x_{i} & 0\\ 0 & 1 \end{array}\right)\right), \end{array}$$

with $\left(y_{1},x_{1}\right),\ldots ,\left(y_{n},x_{n}\right)$ the point of contamination. We next derive the expression of the IF for the Wald-type tests presented in Section 3. The statistical functional associated with the Wald-type tests for the linear null hypothesis (11) at the distributions $G=\left(G_{1},\ldots ,G_{n}\right)$, ignoring the constant n, is given by

(16) $$W_{\alpha }\left(G\right)={\left(M^{T}T_{\alpha }\left(G\right)-m\right)}^{T}{\left(M^{T}A_{\alpha }{\left(T_{\alpha }\left(G\right)\right)}^{-1}M\right)}^{-1}\left(M^{T}T_{\alpha }\left(G\right)-m\right).$$

Again, evaluating the Wald-type test functionals at the contaminated distribution $G_{\varepsilon }$ and implicitly differentiating the expression, we can get the expression of it IF. In particular, the IF of the Wald-type test statistics at the $i_{0}$-th direction and the contamination point $\left(y_{i_{0}},x_{0}\right)$ is given by

$$\begin{array}{cc} IF_{1}\left(\left(y_{i_{0}},x_{0}\right),W_{\alpha },G\right) & ={\left(\frac{\partial W_{\alpha }\left(G_{i_{0},\varepsilon }\right)}{\partial \varepsilon }\right)}_{\varepsilon =0}\\ \; & \;=2{\left(M^{T}T_{\alpha }\left(G\right)-m\right)}^{T}{\left(M^{T}A_{\alpha }{\left(T_{\alpha }\left(G\right)\right)}^{-1}M\right)}^{-1}M^{T}IF\left(\left(y_{i_{0}},x_{0}\right),T_{\alpha },G\right). \end{array}$$

Evaluating the previous expression at the null hypothesis, $M^{T}T_{\alpha }\left(G\right)=m,$ the IF becomes identically zero,

$$IF_{1}\left(\left(y_{i_{0}},x_{i_{0}}\right)W_{\alpha },G\right)=0_{k+1}.$$

Therefore, it is necessary to consider the second order IF of the proposed Wald-type tests. Twice differentiating in $W_{\alpha }\left(G_{\varepsilon }\right)$, we get

$$\begin{array}{cc} IF_{2}\left(\left(y_{i_{0}},x_{i_{0}}\right),W_{\alpha },G\right) & ={\left(\frac{\partial^{2}W_{\alpha }\left(G_{i_{0},\varepsilon }\right)}{\partial \varepsilon^{2}}\right)}_{\varepsilon =0}\\ \; & \;=2IF{\left(\left(y_{i_{0}},x_{i_{0}}\right),T_{\alpha },F_{\beta }\right)}^{T}M{\left(M^{T}A_{\alpha }{\left(T_{\alpha }\left(G\right)\right)}^{-1}M\right)}^{-1}M^{T}IF\left(\left(y_{i_{0}},x_{i_{0}}\right),T_{\alpha },G\right). \end{array}$$

Finally, the second order IF of the Wald-type tests in all directions is given by

$$\begin{array}{cc} IF_{2}\left(\left(y_{1},x_{1}\right),\ldots ,\left(y_{n},x_{n}\right),W_{\alpha },G\right)\; & ={\left(\frac{\partial^{2}W_{\alpha }\left(G_{\varepsilon }\right)}{\partial \varepsilon^{2}}\right)}_{\varepsilon =0}\\ \; & \;=2IF{\left(\left(y_{1},x_{1}\right),\ldots ,\left(y_{n},x_{n}\right),T_{\alpha },G\right)}^{T}M{\left(M^{T}A_{\alpha }{\left(T_{\alpha }\left(G\right)\right)}^{-1}M\right)}^{-1}M^{T}\\ \; & \;\cdot IF(\left(y_{1},x_{1}\right),\ldots ,\left(y_{n},x_{n}\right),T_{\alpha },G). \end{array}$$

**Figure 1:**
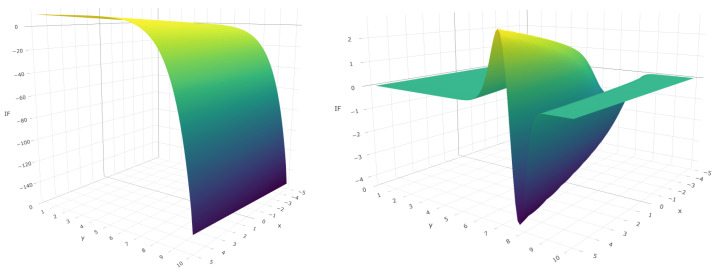
IF of MRPEs with α=0 (**left**) and α=0.5 (**right**) of Poisson regression model.

To asses the robustness of the MRPEs and Wald-type test statistics we must discuss the boundedness of the corresponding IF. The boundedness of the second order IF of the Wald-type test statistics is determined by the boundedness of the IF of the MRPEs. Further, the matrix $\Psi_{n}\left(\gamma \right)$ is assumed to be bounded, so the robustness of the estimators only depend on the second factor of the IF. Most standard GLMs enjoy such properties for positives values of α, but the influence function is unbounded at α=0, corresponding with the MLE. As an illustrative example, Figure 1 plots the IF of the MRPEs for the Poisson regression model with different values of α=0,0.5 at one direction. The model is fitted with only one covariate, the parameter ϕ is known for Poisson regression (ϕ=1) and the true regression vector is fixed β=1. As shown, the IF of the MRPEs with positives values of α are bounded, whereas the IF of the MLE is not, indicating it lack of robustness.

## 5. Numerical Analysis: Poisson Regression Model

We illustrate the proposed robust method for the Poisson regression model. As pointed out in Section 1 the Poisson regression model belongs to the GLM with known shape parameter ϕ=1, location parameter $\theta_{i}=x_{i}^{T}\beta$ and known functions $b\left(\theta_{i}\right)=\exp\left(x_{i}^{T}\beta \right)$ and $c\left(y_{i}\right)=-\log\left(y_{i}!\right)$. Since the nuisance parameter is known, for the seek of simplicity in the following we only use β=γ. In Poisson regression, the mean of the response variable is linked to the linear predictor through the natural logarithm, i.e., $\mu_{i}=\exp\left(x_{i}^{T}\beta \right).$ Thus, we can apply the previous proposed method to estimate the vector of regression parameters β with objective function given in Equation (5).

The results provided are computed in the software R. The minimization of the objective function is performed using the implemented *optim()* function, which applies the Nelder–Mead iterative algorithm (Nelder and Mead [32]). Nelder–Mead optimization algorithm is robust although relatively slow. The corresponding objective function $T_{n}^{\alpha }\left(\gamma \right)$ given in (5) is highly nonlinear and requires the evaluation of nontrivial quantities. Further, the computation of the Wald-type test statistics defined in (13) requires to evaluate the covariance matrix of the MRPEs, involving nontrivial integrals. Some simplified expressions of the main quantities defined throughout the paper for the Poisson regression model, such as $L_{\alpha }^{i}\left(\beta \right),K_{1i}\left(y,\beta \right),N_{i}\left(y,\beta \right),m_{1i}\left(\beta \right),m_{11i}\left(\beta \right)$ or $l_{11i}\left(\beta \right),$ are given in the Appendix B. There is no closed expression for these quantities, and they need to be approximated numerically. Since the minimization is iteratively performed, computing such expressions at each step of the algorithm and for each observation may entail an increased computational burden. Nonetheless, the complexity is not significant for low-dimensional data. On the other hand, the optimum in (5) need not to be uniquely defined, since the objective function may have several local minima. Then, the choice of the initial value of the iterative algorithm is crucial. Ideally, a good initial point should be consistent and robust. In our results the MLE is used as initial estimate for the algorithm.

We analyze the performance of the proposed methods in Poisson regression through a simulation study. We asses the behavior of the MRPE under the sparse Poisson regression model with k=12 covariates but only 3 significant variables. We set the 12-dimensional regression parameter β=(1.8,1,0,0,1.5,0,…0) and we generate the explanatory variables, $x_{i}$, from the standard uniform distribution with variance-covariance matrix having Toeplitz structure, with the (j,l)-th element being $0.5^{\left|j-l\right|},j,l=1,\ldots ,p$. The response variables are generated from the Poisson regression model with mean $\mu_{i}=x_{i}^{T}\beta ,$$Y_{i}∼P\left(\mu_{i}\right).$ To evaluate the robustness of the proposed estimators, we contaminate the responses using a perturbed distribution of the form $\left(1-b\right)P\left(\mu_{i}\right)+bP\left(2\mu_{i}\right),$ where *b* is a realization of a Bernoulli variable with parameter ε so called the contamination level. That is, the distribution of the contaminated responses lies in a small neighbourhood of the assumed model. We repeat the process R=1000 for each value of α.

**Figure 2:**
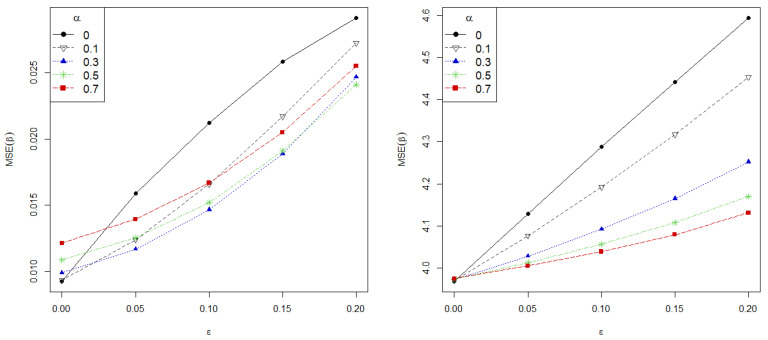
Mean Squared Error (MSE) on estimation (**left**) and prediction (**right**) against contamination level on data.

Figure 2 presents the mean squared error of the estimate (MSE), $\mathrm{MSE}=\left|\right|{\hat{\beta }}_{\alpha }-\beta {\left|\right|}_{2},$ (left) and the MSE on the prediction (right) against contamination level on data for different values of α=0,0.1,0.3,0.5 and 0.7. The sample size is fixed at n=200 and the MSE on the prediction is calculated using n=200 new observations following the true model. As shown, greater values of α correspond to more robust estimators, revealing the role of the tuning parameter on the robustness gain. Most strikingly, the MSE grows linearly for the MLE, while the proposed estimators manage to maintain a low error in all contaminated scenarios.

**Figure 3:**
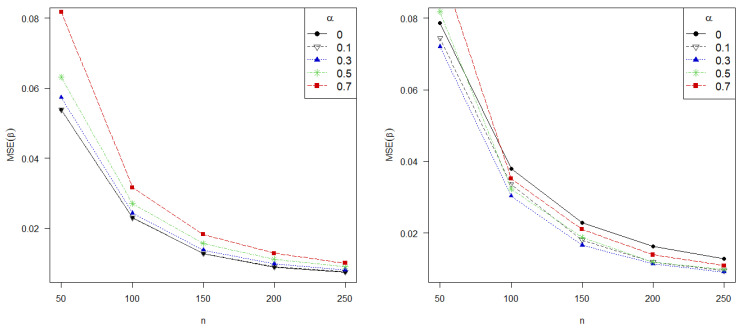
MSE in estimation of β in absence of contamination (**left**) and under 5% of contamination level in data (**right**) with different values of α against sample size for Poisson regression model.

Furthermore, it is to be expected that the error of the estimate decreases with larger samples sizes. In this regard, Figure 3 shows the MSE for different values of α=0,0.1,0.3,0.5 and 0.7, against the sample size in the absence of contamination (left) and under 5% of contamination. Our proposed estimators are more robust than the classical MLE with almost all contaminated scenarios, since the MSE committed is lower for all positives values of α than for α=0 (corresponding to the MLE), except for too small sample sizes. Conversely, the MLE is, as expected, the most efficient estimator in absence of contamination, closely to our proposed estimators with α=0.1,0.3, highlighting the importance of α in controlling the trade-off between efficiency and robustness. In this regard, values of α about 0.3 perform the best taking into account the low loss of efficiency and the gain in robustness. Finally, note that small sample sizes adversely affect to greater values of α.

On the other hand, one could be interested on testing the significance of the selected variables. For this purpose, we simplify the true model and we examine the performance of the proposed Wald-type test statistics under different true coefficients values. In particular, let us consider a Poisson regression model with only two covariates, generated from the uniform distribution as before, and the linear null hypothesis

(17) $$H_{0}:\beta_{2}=0.$$

That is, we are interested in assessing the significance of the second variable. The sample size if fixed at n=200 and the true value of the component of the regression vector is set $\beta_{1}=1.$ We study the power of the tests under increasing signal of the second parameter $\beta_{2}$ and increasing contamination level. Here, the model is contaminated by perturbing the true distribution with $\left(1-b\right)P\left(\mu_{i}\right)+bP\left({\tilde{\mu }}_{i}\right),$ where $\mu_{i}=x_{i}^{T}\beta$ is the mean of the Poisson variable in the absence of contamination, ${\tilde{\mu }}_{i}=x_{i}^{T}\tilde{\beta }$ is the contaminated mean, with $\tilde{\beta }=\left(1,0\right),$ and *b* is a realization of a Bernoulli variable with probability of success ε. Table 1 presents the rejection rate of the Wald-type test statistics for different true values of $\beta_{2}$ under different contaminated scenarios. As expected, stronger signals produce higher power for all Wald-type test. Moreover, the power of the Wald-type test statistics based on the MLE decreases when increasing the contamination, whereas the power of the statistics based on the MRPEs with positives values of α keeps sufficiently high. Then, our proposed robust estimators are able to detect the significance of the variable even in heavily contaminated scenarios.

## 6. Real Data Applications

### 6.1. Example I: Poisson Regression Regression

We finally apply our proposed estimators in a real dataset arising from Crohn’s disease. The data were first studied in Lô and Ronchetti [33] to asses the adverse events of a drug. The clinical study included 117 patients affected by the disease, for whom information was recorded for 7 explanatory variables: BMI (body mass index), HEIGHT, COUNTRY (one of the two countries where the patient lives), SEX, AGE, WEIGHT, and TREAT (the drug taken by the patient in factor form: placebo, Dose 1, Dose 2), in addition to the response variable AE (number of adverse events). Lô and Ronchetti [33] considered a Poisson regression model for the Crohn data and determined that only variables Dose 1, BMI, HEIGHT, SEX, AGE, and COUNTRY may be essentially significant. Further, they flagged observations 23rd, 49th, and 51st to be highly influential on the classical analysis. Table 2 presents the estimated coefficient of the explanatory variable when fitting the Poisson regression model. Robust methods suggest higher coefficients for the variables BMI and AGE, whereas fewer values for the coefficients of the categorical variables COUNTRY, SEX, Dose 1.

Following the discussion in Lô and Ronchetti [33], classical tests may not select variable AGE to be significant. Then, we propose testing the significance of that variable using Wald-type test statics based on different values α. Table 3 shows the p-values of the corresponding tests with null hypothesis $H_{0}$: AGE = 0, with the original data and after removing the outlying observations.

The MLE rejects the significance of the variable AGE when the original data are used, whereas the Wald-type test statistics with positives values of α indicate strong evidence against the null hypothesis. In contrast, if the influential observations are removed, all Wald-type test statistics agree in the significance of the variable. This example illustrates the robustness of the proposed statistics.

### 6.2. Example II: Binomial Regression

We finally illustrate the applicability of the MRPE for robust inference in the binomial regression model. We examine the damaged carrots dataset, first studied in Phelps [34] and later discussed by Cantoni and Ronchetti [8] and Ghosh and Basu [13] to illustrate robust procedures for binomial regression. The data contain 24 samples, among which the 14th observation was flagged as an outlier in the y-space but not a leverage point. The data are issued from a soil experiment and give the proportion of carrots showing insect damage in a trial with three blocks and eight dose levels of insecticide. The explanatory variables are the logarithm transform of the dose (Logdose) and two dummy variables for Blocks 1 and 2.

Binomial regression is a natural extension of the logistic regression when the response variable *Y* does not follow a Bernoulli distribution but a Binomial distribution counting the number of successes in a series of *m* independent Bernoulli trials. Binomial regression model belongs to the GLM with known shape parameter ϕ=1, location parameter $\theta_{i}=x_{i}^{T}\beta$ and functions $b\left(\theta_{i}\right)=m\log\left(1+\exp(x_{i}^{T}\beta )\right)$ and c(yi)=log(myi). The mean of the response variable is then linked to the linear predictor through the logit function, i.e.,

$$\log\left(\frac{\mu_{i}}{m-\mu_{i}}\right)=x_{i}^{T}\beta .$$

Table 4 presents the estimated coefficients of the regression vector for the carrots data using the MLE and robust MRPEs when the model is fitted with the original data and the model fitted without the outlying observation. The results provided are computed in the same manner as in Section 5, adapting the corresponding quantities in Equation (5) for the binomial model. All integrals involved were numerically approximated, and the MLE is used as initial estimate for the optimization algorithm. The influence of observation 14 stands out when using the MLE; the estimated coefficients are remarkably different when fitting the model with and without observation 14. In contrast, all methods estimate similar coefficients after removing the outlying observation, coinciding with the robust estimates for moderately high values of the tuning parameter α.

## 7. Conclussions

In this paper, we presented the MRPE and Wald-type test statistics for GLMs. The proposed MRPEs and statistics have appealing robustness properties where the data are contaminated due to outliers or leverage points. MRPEs are consistent and asymptotically normal and represent an attractive alternative to the classical nonrobust methods. Additionally, robust Wald-type test statistics, based on the MRPEs, were developed. Through the study of the IFs and the development of an extensive simulation study, we proved their robustness from a theoretical and practical point of view, respectively. In particular, we illustrated the superior performance of the MRPEs and the corresponding Wald-type tests for the Poisson regression model.

## Figures and Tables

**Table 1:** Rejection rate of Wald-type test statistics based on MRPEs with different true values of $\beta_{2}$ and contamination levels.

$\beta_{2}$	α	Contamination Level					
		0	5%	10%	15%	20%	25%
0.3	0	0.332	0.264	0.227	0.187	0.157	0.141
	0.1	0.435	0.376	0.328	0.285	0.251	0.223
	0.3	0.557	0.511	0.483	0.416	0.390	0.360
	0.5	0.617	0.563	0.533	0.493	0.467	0.427
	0.7	0.638	0.590	0.568	0.536	0.513	0.476
0.5	0	0.756	0.730	0.683	0.621	0.551	0.493
	0.1	0.833	0.798	0.775	0.736	0.681	0.622
	0.3	0.885	0.870	0.864	0.829	0.792	0.752
	0.5	0.895	0.891	0.886	0.867	0.842	0.814
	0.7	0.901	0.897	0.893	0.879	0.854	0.832
0.7	0	0.971	0.979	0.968	0.948	0.915	0.862
	0.1	0.980	0.988	0.983	0.973	0.962	0.932
	0.3	0.988	0.995	0.992	0.987	0.985	0.969
	0.5	0.989	0.995	0.995	0.992	0.992	0.977
	0.7	0.989	0.995	0.993	0.995	0.990	0.983

**Table 2:** Estimated coefficients for Crohn’s disease data for different values of α with original data and clean data (after removing influential observations).

	Intercept	BMI	Height	Age	Country	Sex	Dose 1
**Original Data**							
MLE (α= 0)	6.261	0.026	−0.037	0.012	−0.394	−0.646	−0.533
α= 0.1	5.197	0.037	−0.033	0.014	−0.489	−0.800	−0.469
α= 0.3	4.798	0.058	−0.036	0.021	−0.545	−1.284	−0.832
α= 0.5	4.391	0.067	−0.037	0.028	−0.557	−1.535	−1.036
α= 0.7	5.699	0.067	−0.047	0.036	−0.737	−1.759	−1.157

**Table 3:** *p*-values of test with null hypothesis $H_{0}$: AGE = 0 with original and clean data (after removing influential observations).

	Original Data	Clean Data
MLE (α= 0)	0.059	0.011
α= 0.1	0.018	0.004
α= 0.3	0.001	0.000
α= 0.5	0.000	0.000
α= 0.7	0.000	0.000

**Table 4:** Estimated coefficients for damaged carrots data for different values of α with original data and clean data (after outliers removal).

	Intercept	Logdose	B1	B2
**Original Data**				
MLE (α=0)	1.480	−1.817	0.542	0.843
α= 0.1	1.729	−1.949	0.527	0.755
α= 0.3	2.017	−2.100	0.479	0.652
α= 0.5	2.090	−2.134	0.386	0.625
α= 0.7	2.150	−2.161	0.258	0.615
**Clean Data**				
MLE (α=0)	2.141	−2.179	0.546	0.636
α= 0.1	2.126	−2.167	0.529	0.633
α= 0.3	2.105	−2.149	0.479	0.627
α= 0.5	2.108	−2.144	0.385	0.621
α= 0.7	2.154	−2.163	0.257	0.614

## Data Availability

The real datasets are publicly available on the R package *robustbase* in CRAN under the names of *CrohnD* (Poisson regression example) and *carrots* (binomial regression example).

## Abbreviations

The following abbreviations are used in this manuscript:

DPD	Density Power Divergence
IF	Influence Function
GLM	Genelarized Linear Model
LRM	Linear Regression Model
MLE	Maximum Likelihood Estimator
MRPE	Minimum Rényi Pseudodistance Estimator
RP	Rényi Pseudodistance

## Appendix A. Proof of Theorem 3

Let us define

$$l_{\eta }\left(\zeta \right)={\left(M^{T}\eta -m\right)}^{T}{\left(M^{T}A_{\alpha }{\left(\zeta \right)}^{-1}M\right)}^{-1}\left(M^{T}\eta -m\right)$$

so the Wald-type test statistic is such that

$$nl_{{\hat{\gamma }}_{\alpha }}\left({\hat{\gamma }}_{\alpha }\right)=W_{n}\left({\hat{\gamma }}_{\alpha }\right).$$

We know that ${\hat{\gamma }}_{\alpha }\underset{n\to \infty }{\overset{P}{\to }}\gamma_{1}$ and therefore $l_{{\hat{\gamma }}_{\alpha }}\left(\gamma_{1}\right)$ and $l_{\gamma_{1}}\left(\gamma_{1}\right)$ have the same asymptotic distribution. A first order Taylor expansion of $g\left(\zeta \right)=l_{{\hat{\gamma }}_{\alpha }}\left(\zeta \right)$ at ${\hat{\gamma }}_{\alpha }$ around $\gamma_{1}$ gives,

$$l_{{\hat{\gamma }}_{\alpha }}\left({\hat{\gamma }}_{\alpha }\right)=l_{{\hat{\gamma }}_{\alpha }}\left(\gamma_{1}\right)+{\left(\frac{\partial l_{{\hat{\gamma }}_{\alpha }}\left(\zeta \right)}{\partial \zeta^{T}}\right)}_{\gamma =\gamma_{1}}\left({\hat{\gamma }}_{\alpha }-\gamma_{1}\right)+o_{p}\left(∥{\hat{\gamma }}_{\alpha }-\gamma_{1}∥\right).$$

Based on the asymptotic distribution of ${\hat{\gamma }}_{\alpha }$ we have

$$\sqrt{n}o_{p}\left(∥{\hat{\gamma }}_{\alpha }-\gamma_{1}∥\right)=o_{p}\left(1\right)$$

therefore

$$\sqrt{n}\left(l_{{\hat{\gamma }}_{\alpha }}\left({\hat{\gamma }}_{\alpha }\right)-l_{\gamma_{1}}\left(\gamma_{1}\right)\right)\;\mathrm{and}\;\sqrt{n}{\left(\frac{\partial l_{{\hat{\gamma }}_{\alpha }}\left(\zeta \right)}{\partial \zeta^{T}}\right)}_{\gamma =\gamma_{1}}\left({\hat{\gamma }}_{\alpha }-\gamma_{1}\right)$$

have asymptotically the same distribution, i.e.,

$$\sqrt{n}\left(l_{{\hat{\gamma }}_{\alpha }}\left({\hat{\gamma }}_{\alpha }\right)-l_{\gamma_{1}}\left(\theta_{1}\right)\right)\underset{n\to \infty }{\overset{L}{\to }}N\left(0_{k+1},{\left(\frac{\partial l_{{\hat{\gamma }}_{\alpha }}\left(\zeta \right)}{\partial \zeta^{T}}\right)}_{\gamma =\gamma_{1}}A_{\alpha }{\left(\gamma_{1}\right)}^{-1}{\left(\frac{\partial l_{{\hat{\gamma }}_{\alpha }}\left(\zeta \right)}{\partial \zeta }\right)}_{\gamma =\gamma_{1}}\right).$$

Now, we shall denote,

$$\sigma^{2}\left(\gamma_{1}\right)={\left(\frac{\partial l_{{\hat{\gamma }}_{\alpha }}\left(\zeta \right)}{\partial \zeta^{T}}\right)}_{\gamma =\gamma_{1}}A_{\alpha }{\left(\gamma_{1}\right)}^{-1}{\left(\frac{\partial l_{{\hat{\gamma }}_{\alpha }}\left(\zeta \right)}{\partial \zeta }\right)}_{\gamma =\gamma_{1}}.$$

Then, we have,

$$\begin{array}{cc} P_{\gamma_{1}}\left(W_{n}\left({\hat{\gamma }}_{\alpha }\right)>\chi_{r,\alpha }^{2}\right) & =P_{\gamma_{1}}\left(W_{n}\left({\hat{\gamma }}_{\alpha }\right)-nl_{\gamma_{1}}\left(\gamma_{1}\right)>\chi_{r,\alpha }^{2}-nl_{\gamma_{1}}\left(\gamma_{1}\right)\right)\\ \; & =P_{\gamma_{1}}\left(\frac{\sqrt{n}}{\sigma \left(\gamma_{1}\right)}\left(l_{{\hat{\gamma }}_{\alpha }}\left({\hat{\gamma }}_{\alpha }\right)-l_{\gamma_{1}}\left(\gamma_{1}\right)\right)>\frac{1}{\sigma \left(\gamma_{1}\right)}\left(\frac{\chi_{r,\alpha }^{2}}{\sqrt{n}}-\sqrt{n}l_{\gamma_{1}}\left(\gamma_{1}\right)\right)\right)\\ \; & \approx 1-\phi_{N(0,1)}\left(\frac{1}{\sigma \left(\gamma_{1}\right)}\left(\frac{\chi_{r,\alpha }^{2}}{\sqrt{n}}-\sqrt{n}l_{\gamma_{1}}\left(\gamma_{1}\right)\right)\right), \end{array}$$

where $\phi_{N(0,1)}\left(t\right)$ represents the distribution function of a standard normal distribution evaluated at t. Finally,

$$\lim_{n\to \infty }P_{\gamma_{1}}\left(W_{n}\left({\hat{\gamma }}_{\alpha }\right)>\chi_{r,\alpha }^{2}\right)=1.$$

## Appendix B. Poisson Regression Model

We derive here some explicit expression for the particular case of the Poisson regression. Following the discussion in Section 5, we denote here γ=β since the nuisance parameter is known, ϕ=1. The Poisson distribution with parameter $e^{x_{i}^{T}\beta }$ is given by

$$f_{i}\left(y,\beta \right)=\frac{1}{y!}e^{-e^{x_{i}^{T}\beta }}e^{yx_{i}^{T}\beta },y=0,1,\ldots .$$

Differentiating its logarithm with respect to the regression vector, we get

$$\frac{\partial \log f_{i}\left(y,\beta \right)}{\partial \beta }=\left(y-e^{x_{i}^{T}\beta }\right)x_{i}^{T}.$$

so we can write

$$K_{1i}\left(y,\beta \right)=y-e^{x_{i}^{T}\beta }.$$

Further, we have that

$$\begin{array}{c} N_{i}\left(y,\beta \right)=\frac{f_{i}{\left(y,\beta \right)}^{\alpha }}{\sum_{y=0}^{\infty }f_{i}{\left(y,\beta \right)}^{\alpha +1}}\sum_{y=0}^{\infty }f_{i}{\left(y,\beta \right)}^{\alpha +1}\left(y-e^{x_{i}^{T}\beta }\right). \end{array}$$

so the estimating equations of the Poisson regression model are given by

(A1) $$\sum_{i=1}^{n}\frac{1}{L_{\alpha }^{i}\left(\beta \right)}\left(f_{i}{\left(y_{i},\beta \right)}^{\alpha }\left(y_{i}-e^{x_{i}^{T}\beta }\right)-N_{i}\left(y_{i},\beta \right)\right)x_{i}=0_{k}.$$

For α=0, we have

$$N_{i}\left(y_{i},\beta \right)=0\;\mathrm{and}\;L_{\alpha }^{i}\left(\beta \right)=1$$

so the estimating equations are given by

$$\sum_{i=1}^{n}\left(y_{i}-e^{x_{i}^{T}\beta }\right)x_{i}=0_{k},$$

yielding to the maximum likelihood estimating equations.

On the other hand, the asymptotic distribution of ${\hat{\beta }}_{\alpha }$ is given by

$$\sqrt{n}{\left(X^{T}D_{11}X\right)}^{-\frac{1}{2}}\frac{1}{n}X^{T}\left(D_{11}^{*}-{\left(D_{1}^{*}\right)}^{T}D_{1}^{*}\right)X\left({\hat{\beta }}_{\alpha }-\beta \right)\underset{n\to \infty }{\overset{L}{\to }}N\left(0_{k},I_{k}\right)$$

being

$$D_{11}=diag\left(l_{11i}\left(\beta \right)\right),$$

with

$$l_{11i}\left(\beta \right)=\frac{1}{L_{\alpha }^{i}{\left(\beta \right)}^{2}}\sum_{y=0}^{\infty }f_{i}{\left(y,\beta \right)}^{2\alpha +1}\left(K_{1i}\left(y,\beta \right)-m_{1i}\left(\beta \right)\right)$$

and

$$m_{1i}\left(\beta \right)=\frac{1}{\sum_{y=0}^{\infty }f_{i}{\left(y,\beta \right)}^{\alpha +1}}\sum_{y=0}^{\infty }f_{i}{\left(y,\beta \right)}^{\alpha +1}\left(y-e^{x_{i}^{T}\beta }\right).$$

Finally

$$\begin{array}{cc} D_{11}^{*} & =diag(m_{11i}\left(\left(\beta \right)\right))=\frac{1}{\sum_{y=0}^{\infty }f_{i}{\left(y,\beta \right)}^{\alpha +1}}\sum_{y=0}^{\infty }f_{i}{\left(y,\beta \right)}^{\alpha +1}{\left(y-e^{x_{i}^{T}\beta }\right)}^{2}. \end{array}$$
